# Misunderstanding publication bias: editors are not blameless after all

**DOI:** 10.12688/f1000research.1-59.v1

**Published:** 2012-12-04

**Authors:** Stephen Senn

**Affiliations:** 1 Competence Center for Methodology and Statistics, Centre de Recherches Sante, Strassen, Luxembourg

## Abstract

In analysing whether there is an editorial bias in favour of positive studies, researchers have made implicit assumptions that are implausible. In particular, to justify the conclusion that there is no bias because observed editorial acceptance rates do not favour positive studies, the assumption that the decision to submit an article is based solely on quality would be required. If, on the other hand, submission were based on perceived probability of acceptance, negative and positive studies would not differ in terms of acceptance rates, but in terms of quality.

It is shown, using a simple graphical model, how similar underlying situations as regards the relationship between quality and probability of acceptance on the one hand and study outcome (positive or negative) and probability of acceptance on the other could produce dramatically different results depending on the behaviour of authors.

Furthermore, there is, in fact, some evidence that submitted negative studies are, on average, of higher quality than positive ones. This calls into question the standard interpretation of the studies examining editorial bias. It would appear that despite similar probabilities of acceptance for negative and positive studies, editors could be discriminating against negative studies.

## Bad JAMA?

In his recent book *Bad Pharma* ^1^, Ben Goldacre dismisses the possibility that the reason that many negative studies are unpublished is because there is an editorial bias in favour of positive and against negative studies. He cites a number of papers ^2– 5^ in support of this, writing: "Here again the journals seem blameless: 745 manuscripts submitted to the *Journal of the American Medical Association* ( *JAMA*) were followed up, and there was no difference in acceptance and non-significant findings. The same thing has been tried with papers submitted to the *BMJ, The Lancet, Annals of Internal Medicine* and the *Journal of Bone and Joint Surgery*. Again and again no effect was found". (p34).

A very thorough meta-analysis carried out by Song *et al.* ^6^, which provides a useful summary of the studies that Goldacre cites, would appear to justify Goldacre’s conclusion. Song *et al.* find an overall odds ratio in favour of positive studies of 1.06, with confidence limits of 0.8 to 1.39, writing: "After the submission of a manuscript for publication, editorial decisions were not clearly associated with study results" (p10). The wording is more circumspect than Goldacre’s, but the sentiment seems broadly the same.

However, as I explain below, these studies are inherently incapable of showing that there is no publication bias by journals and in fact, properly interpreted, they provide evidence that could be compatible with the opposite of what Goldacre and others claim.

## Quality matters

**Figure 1.**
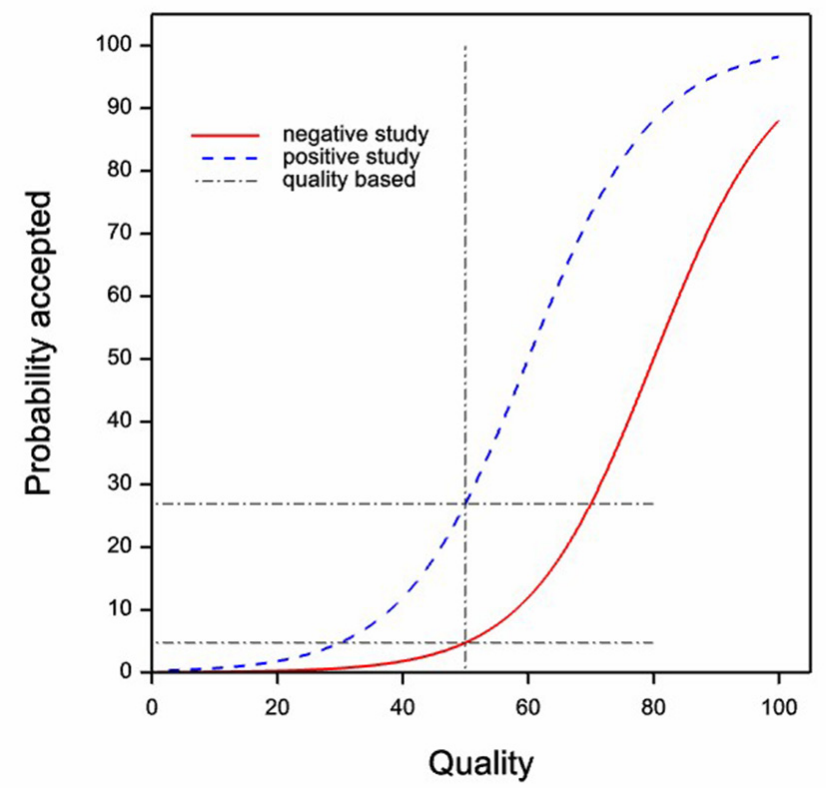
The situation that would apply regarding acceptance of papers were authors to submit to journals based on the quality of the research undertaken and given that there was discrimination by editors in favour of positive studies. The acceptance curve for negative studies is shown in solid red and that for positive studies in dashed blue. The vertical dashed line shows a postulated *quality* threshold for submission and the horizontal dashed lines indicate the acceptance probabilities that would result.

Figure 1 shows two imaginary curves for a given journal illustrating the probability of acceptance for a manuscript submitted to a journal as a function of the quality (measured on an arbitrary scale from 0 to 100) of that manuscript. In the figure it is assumed that the probability of acceptance for a manuscript of a given quality is always higher for a positive study than for a negative one.

The curves are not meant to be realistic in detail, but to suffice conceptually to represent the idea that higher quality increases the probability of acceptance (programmed in GenStat: see 'Publish or perish GenStat program'). Of course, the reader could always object that the curves do not represent what he or she considers the relationship between quality and probability of acceptance might be. However, the point is that the details of the precise shape of the curves are irrelevant provided only that two features survive: first that other things being equal higher ‘quality’ leads to higher probability of acceptance and second, other things being equal, that positive studies are more likely to be accepted than negative ones. We can refer to the two curves in Figure 1 as *quality acceptance curves*. Now, consider two assumptions. First, that authors make a decision to submit to a given journal in their field based on quality and not on whether the study is positive or negative. Call this the *equal quality assumption*. Second, that the distribution of positive and negative studies by quality is equal. Call this the *identical distribution assumption*. If these assumptions are true, then a lack of observed difference in probabilities of acceptance is indicative that the two quality acceptance curves are the same and this would imply that studies of a given quality have the same chance of being published whether negative or positive: In other words, that comparing like with like, there was no bias in favour of positive studies.

Publish or perish GenStat program To illustrate possible issues regarding probability of a paper being published

The quality assumption is illustrated in Figure 1. Here a vertical *quality submission threshold* is shown. Papers below the threshold (to the left of the boundary) would not be submitted but those above (to the right) will be. The figures show the probability of acceptance *at the threshold* for the two types of study, and these are very different, being about 5% for negative studies and 27% for positive ones.

## Probability matters

**Figure 2.**
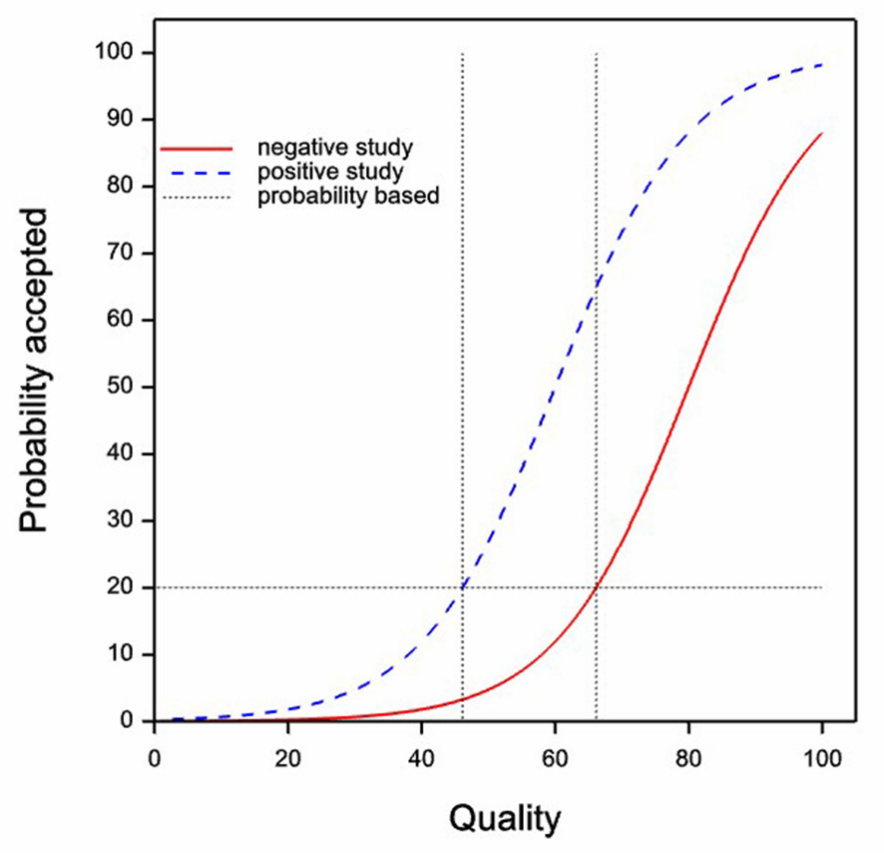
The same quality acceptance curves as in Figure 1. This time however, it is supposed that authors make their decision to submit based on probability of acceptance. As before, the solid red curve gives the probability of acceptance for negative studies and the dashed blue curve for positive studies. The horizontal dashed line shows a postulated *probability* threshold for submission and the vertical dashed lines indicate the quality thresholds that would result.

However, if authors act rationally, setting a quality submission threshold is not, except indirectly, how they will judge whether or not to submit a manuscript to a given journal. Self-interest would require them to take into account the cost of submission (in terms of effort), the reward if published (in terms of kudos and so forth) and the probability of acceptance. Here, an alternative threshold might be supposed. We might imagine, other things being equal, that authors employ a *probability submission threshold*. We can call the assumption corresponding to this the *equal probability assumption*. In that case what might apply is the situation shown in Figure 2. Now we have a horizontal probability threshold. Now it is the case that authors will not submit papers unless the probability of acceptance is at least equal to 20%. However, this threshold probability does *not* differ between negative and positive studies and the fact that it does not differ does not show a lack of bias. *It is now the quality that differs at the probability threshold not the probability that differs at the quality threshold*. Negative studies will have to be of higher quality to be worth submitting. In fact, the quality at the threshold for positive studies is about 46, whereas for negative studies it is 66. Again, whether this would translate into observed identical probabilities of acceptance depends on whether the mixture of studies submitted is identical.

## Ps or Qs?

Therefore, in judging the evidence, a key issue is which of the two assumptions, the equal quality assumption of Figure 1 or the equal probability assumption of Figure 2 is more relevant (there is still, of course, the issue of the identical distribution assumption, to be discussed below). One might argue that one has no more reason to suppose that the equal probability assumption is true than the equal quality assumption.

However, this would be to ignore the evidence of a paper Goldacre cites ^3^. This is by Lynch *et al.* and has a most revealing title ‘Commercially funded and United States-based research is more likely to be published; good-quality studies with negative outcomes are not’ and the abstract states, ‘Studies with a positive outcome were no more likely to be published than were those with a negative outcome (p=0.410) …studies with a negative outcome were of higher quality (p=0.003)’. In other words this paper provides evidence compatible with Figure 2.

## In conclusion: caution is called for

All too easily we make the unconscious assumption that data we have seen have arisen in an inferentially neutral way so that from the point of study onwards, their message can be read at face value. However, studying papers submitted to journals is not like studying patients who have been randomised to treatment in a clinical trial. In fact, had researchers paused to think about it, they would have realised that the quality assumption was implausible. Goldacre himself points out that many studies are not submitted and it seems that negative studies are less likely to be submitted. That being so, it seems almost impossible to believe that the mixture by quality of submitted positive and negative studies could be identical. It would then follow that even if we could satisfy ourselves as to which of the quality or distributional assumptions were more reasonable, it would be difficult to know how this would pan out in terms of crude acceptance probabilities.

A further difficulty is that it also seems plausible that higher quality studies are more likely to lead to a positive result. Certainly, to believe the contrary, is very unpalatable to a statistician like myself. (Of course, one could imagine a world in which researchers were naturally tending to produce studies that were larger than they needed to be but were persuaded by statisticians to get by with fewer subjects. In that case the difference a statistician would make would be mainly in reducing cost and not in increasing the probability of a positive result, but my personal experience, at least, does not support this).

In conclusion, I consider that the prospects for disentangling cause and effect when it comes to publication bias are not great. Certainly I do not think that the studies carried out so far can be taken as proof that editors have no bias in favour of positive studies. On the contrary, a very plausible explanation is the authors believe that editors are biased in favour of positive studies and are right.
