# Supplementary material for: Misunderstanding publication bias: editors are not blameless after all
Source: F1000Res. 2012 Dec 4;1:59. [Version 1] doi: 10.12688/f1000research.1-59.v1 (PMC3782365; doi:10.12688/f1000research.1-59.v1)
Supplement: Peer review history

# Peer review: Referee response for version 1

**Published:** 2012-12-10

**Authors:** Vance Berger

**Affiliations:** 1 Biometry Research Group, National Cancer Institute, National Institutes of Health (NIH), Rockville, MD, USA

Normally when reviewing I like to add something novel, with some added value. But in this case, I have nothing to add. The paper is complete as it is; the point is a valid one, and it was made very well.

I have read this submission. I believe that I have an appropriate level of expertise to confirm that it is of an acceptable scientific standard.

# Peer review: Referee response for version 1

**Published:** 2012-12-07

**Authors:** James Roger

**Affiliations:** 1 Research Statistics Unit, GlaxoSmithKline, Brentford, UK

This is an interesting opinion piece that suggests authors of scientific papers, may through their decision to submit, be inducing a correlation between the quality of paper and whether the results are negative or positive.

It also suggests there may be an inherent tendency for better quality research to come up with truly positive results. So there is possibly an association between quality and positiveness of results in papers submitted to journals. If so, then we would expect to see papers selected on quality showing more positive results than those rejected. The lack of such an association suggests there may indeed be something amiss, but hardly anything more than that.

A suggestion for the author is to change the title to: ‘Misunderstanding publication bias: editors are perhaps not blameless after all’.

I have read this submission. I believe that I have an appropriate level of expertise to confirm that it is of an acceptable scientific standard.
